# Effect of *Lactobacillus rhamnosus* on the development of B cells in gut‐associated lymphoid tissue of BALB/c mice

**DOI:** 10.1111/jcmm.15574

**Published:** 2020-07-08

**Authors:** Chun‐wei Shi, Yan Zeng, Gui‐lian Yang, Yan‐long Jiang, Wen‐tao Yang, Yi‐qiu Chen, Jing‐ying Wang, Jian‐zhong Wang, Yuan‐huan Kang, Hai‐bin Huang, Li‐ping Ye, Xin Cao, Chun‐feng Wang

**Affiliations:** ^1^ College of Animal Science and Technology Jilin Provincial Engineering Research Center of Animal Probiotics Key Laboratory of Animal Production and Product Quality Safety of Ministry of Education Jilin Agricultural University Changchun China

**Keywords:** activation and antigen presentation, B cell development, immunoglobulin secretion, *Lactobacillus rhamnosus*

Dear Editor,

Lactic acid bacteria (LAB) adhere to the inner surface of gastrointestinal tract and regulate mucosal and systemic immune response through antigen‐presenting cells.^1^ Under the stimulation of immunoglobulin and cytokines, LAB can also affect the regulation of related immune response.^2^ Lactic acid bacteria can inhibit the production of IL‐12 and transcription of IL‐12p40 mRNA by macrophages.^3^ Studies have shown that LAB can induce the production of systemic anti‐inflammatory cytokines, such as interleukin‐10 (IL‐10). Soluble factors produced by LAB inhibit the production of pro‐inflammatory cytokines.^4^, ^5^, ^6^ Oral LAB act on mucosa of gastrointestinal tract, and at least 70% of immune cells are colonized in gut‐associated lymphoid tissue (GALT).^7^ Although bone marrow (BM) is the major primary lymphoid organ of B lymphogenesis for mammals, GALT is also identified as the primary lymphoid organ for B cell development in different species.^8^, ^9^ Besides, microbiota play an essential role for B cell development in mammal GLAT.^9^, ^10^ However, the effect of LAB on the development and function of B cells in GALT needs to be further dissected.

In this study, fifty 1‐week‐old BALB/c mice were randomly divided into two groups, namely the PBS control group and *Lactobacillus rhamnosus* (LGG) group with 25 mice per group. Mice were orally administrated with LGG at the dose of 10^7^ cfu/10 μL every other day for 2 weeks, and mice treated with PBS were used as control. At 7, 14, 21, 28 and 35 days after the treatment, mice were killed for analysing (n = 5 for each time‐point). Firstly, developmental stages of B cells, that is B220^+^CD43^+^IgM^−^IgD^−^ (pro‐B), B220^+^CD43^−^IgM^−^IgD^−^ (pre‐B), B220^+^CD43^−^IgM^+^IgD^−^ (immature B) and B220^+^CD43^−^IgM^+^IgD^+^ (mature B), were detected in mouse BM, intestinal lamina propria (LPL) and Peyer's patches (PPs) by flow cytometry. Mature B cells were analysed in mouse spleen (SPL) and mesenteric lymph nodes (MLN). Secondly, the expression levels of CD40, CD80 and MHC‐Ⅱ on B cells were detected in mouse SPL, MLN and PPs. Lastly, we examined the Secretory Immunoglobulin A (SIgA) level in intestinal lavage fluid and serum IgM, IgA and Immunoglobulin G (IgG) by ELISA.

Figure 1A shows the gating strategy for different developmental stages of B cells. On the 7th day after LGG intervention, the percentage of pro–B cells in BM of LGG group was significantly lower than that of control group, with 1.25 ± 0.17% vs 2.28 ± 0.18% (n = 5; *P* < 0.01, *P* = 0.0033). On the 35th day after LGG intervention, the percentage of pre–B and immature B cells in BM of LGG group decreased significantly compared to control group, with 42.56 ± 6.34% vs 64.64 ± 0.89% for Pre–B cells (n = 5; *P* < 0.01, *P* = 0.0087) and 11.88 ± 3.97% vs 28.46 ± 0.83% for immature B cells (n = 5; *P* < 0.01, *P* = 0.0017), respectively. However, the percentage of mature B cells in BM of LGG group increased significantly compared to control group on the 35th day after LGG intervention, with 21.71 ± 4.19% vs 5.15 ± 0.23% (n = 5; *P* < 0.01, *P* = 0.0043) (Figure 1B). The representative data of B cell development in BM for flow cytometry were shown in Figure S1A‐E. With the cessation of intervention, the percentage of pre–B and immature B cells decreased significantly, while the number of mature B cells increased dramatically in LGG group, indicating that LGG promotes the development of pro‐B to mature B in BM. Similar results were obtained in LPL of intestine, a newly identified primary lymphoid organ for B cell development in mice. On the 21st and 28th days after LGG intervention, the percentage of immature B cells in LPL of LGG group decreased significantly, while the percentage of mature B cells in LPL of LGG group increased significantly compared with control group (Figure 1C). The representative data of B cell development in LPL for flow cytometry were shown in Figure S1F‐J. We also checked the different developmental stages of B cells in PPs. However, LGG intervention had no dramatic effects on B cell progression in PPs, except for some marginal increase of pre–B cell percentage in LGG group on the 21st and 28th days after LGG intervention (Figure 1D; Figure S2). For the secondary lymphoid organ, we found the percentage of mature B cells for LGG group increased in SPL and decreased in MLN compared to control group on the 35th day after LGG intervention, with 60.98 ± 1.26% vs 53.42 ± 0.77% in SPL (n = 5; *P* < 0.01, *P* = .009) and 38.92 ± 2.54% vs 50.28 ± 2.26% in MLN (n = 5; *P* < 0.05, *P* = .0103), respectively (Figure 1E‐F; Figure S3).

**FIGURE 1:**
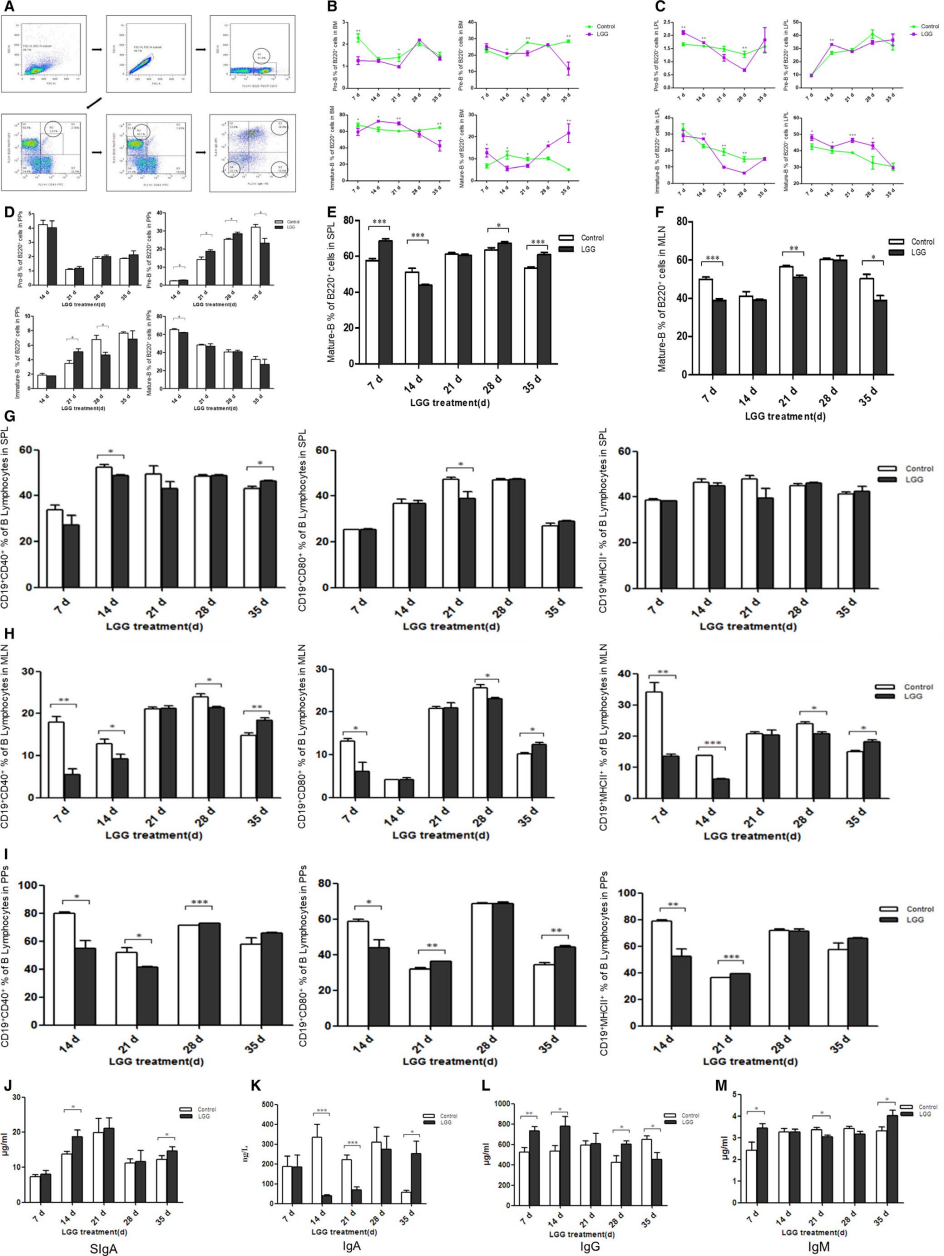
*Lactobacillus rhamnosus* (LGG) intervention can promote the development and function of B lymphocytes. A, Gating strategy for the development and maturation of B lymphocytes. R1 indicated the percentage of total B cell (B220^+^) in the lymphocytes. R2 indicated the percentage of pro–B (B220^+^CD43^+^) cells in the total B lymphocytes. Q4 indicated the percentage of pre–B (B220^+^CD43^−^IgM^‐^IgD^−^) cells in the total B lymphocytes. Q3 indicated the percentage of immature B (B220^+^CD43^−^IgM^+^IgD^−^) cells in the total B lymphocytes. Q2 indicated the percentage of mature B (B220^+^CD43^−^IgM^+^IgD^+^) cells in the total B lymphocytes. B, The proportion changes of pro–B, pre–B, immature B and mature B cells in the B220^+^ cells of BM with the LGG treatment. C, The proportion changes of pro–B, pre–B, immature B and mature B cells in the B220^+^ cells of LPL with the LGG treatment. D, The proportion changes of pro–B, pre–B, immature B and mature B cells in the B220^+^ cells of and Peyer's patches (PPs) with the LGG treatment. E, The proportion changes of mature B cells in the B220^+^ cells of SPL with the LGG treatment. F, The proportion changes of mature B cells in the B220^+^ cells of mesenteric lymph nodes (MLN) with the LGG treatment. G, The proportion changes of CD19^+^ B cell expressing CD40/CD80/MHC‐II among lymphocytes in spleen (SPL) with the LGG treatment. H, The proportion changes of CD19^+^ B cell expressing CD40/CD80/MHC‐II among lymphocytes in MLN with the LGG treatment. I, The proportion changes of CD19^+^ B cell expressing CD40/CD80/MHC‐II among lymphocytes in PPs with the LGG treatment. J, Effect of LGG on the level of SIgA in intestinal lavage fluid of mice. K, Effect of LGG on the level of IgA in serum of mice. L, Effect of LGG on the level of IgG in serum of mice. M, Effect of LGG on the level of IgM in serum of mice. The data were analysed and processed by GraphPad Prism 5.0 software. Student's *t* test was used to compare the data of the two groups, and multiple comparison method of one‐way ANOVA was used to analyse the data of more than two groups. The symbol * indicated *P* < 0.05, ** indicated *P* < 0.01 and *** indicated *P* < 0.001

The expressions of CD40, CD80 and MHC‐Ⅱ on B cell were also determined in SPL, MLN and PPs. The results showed that the treatment with LGG inhibited the expression of CD40, CD80 and MHC‐Ⅱ on B cell surface at the beginning. Along with colonization in the intestine, LGG could increase the expression of CD40, CD80 and MHC‐Ⅱ on B cell surface (Figure 1G‐I; Figure S4). The results suggest that LGG intervention inhibits B cell activation and antigen‐presenting ability to create good conditions for colonization of LGG at the beginning, whereas the stable colonization of LGG in the intestine can promote B cell activation and antigen‐presenting capability.

SIgA level in intestinal lavage fluid and serum IgA, IgG and IgM was analysed by ELISA. LGG could significantly increase the secretion of SIgA in intestinal mucosa on the 14th and 35th days after LGG intervention. At the beginning of LGG intervention, the secretion of IgA in serum of LGG group decreased, while the secretion of IgG and IgM increased compared with control group. With the cessation of intervention, the secretion of IgA and IgM in serum of LGG group increased, while the secretion of IgG decreased compared with control group (Figure 1J‐M). It showed that LGG intervention regulated immunoglobulin secretion of B cell, so as to regulate the mucosal immunity and humoural immunity of mice.

To sum up, LGG intervention can promote the development and maturation of B lymphocytes, enhance the activation and antigen‐presentation ability of B lymphocytes, and regulate the secretion of immunoglobulin by B lymphocytes. Thus, LGG can regulate the mucosal immunity and humoural immunity of mice.

## CONFLICT OF INTEREST

The authors confirm that there are no conflicts of interest.

## AUTHOR CONTRIBUTION

**Chun‐wei Shi:** Formal analysis (lead); Investigation (lead); Methodology (equal); Project administration (equal); Writing‐original draft (equal). **Yan Zeng:** Data curation (equal); Investigation (equal); Methodology (equal); Validation (equal). **Gui‐lian Yang:** Project administration (supporting); Resources (supporting); Supervision (equal). **Yan‐long Jiang:** Investigation (supporting); Methodology (supporting). **Wen‐tao Yang:** Investigation (supporting); Methodology (supporting). **Yi‐qiu Chen:** Investigation (supporting); Methodology (supporting). **Jing‐ying Wang:** Investigation (supporting); Methodology (supporting). **Jian‐zhong Wang:** Investigation (supporting); Methodology (supporting). **Yuan‐huan Kang:** Investigation (supporting); Methodology (supporting). **Hai‐bin Huang:** Investigation (supporting); Resources (supporting). **Li‐ping Ye:** Investigation (supporting); Methodology (supporting). **Xin Cao:** Conceptualization (equal); Funding acquisition (equal); Project administration (lead); Supervision (equal); Writing‐original draft (lead); Writing‐review & editing (equal). **Chunfeng Wang:** Conceptualization (lead); Funding acquisition (lead); Supervision (lead); Writing‐review & editing (equal).

## ETHICAL APPROVAL

All animal experiments were conducted in the Laboratory Animal Center, under the supervision and assessment by the Laboratory Animal Welfare and Ethics Committee of Jilin Agricultural University (No. 2019 04 09 003).

### DATA AVAILABILITY STATEMENT

The data that support the findings of this study are available from the corresponding author upon reasonable request.

REFERENCES1

Iannitti
T
, 
Palmieri
B
. Therapeutical use of probiotic formulations in clinical practice. Clin Nutr. 2010;29(6):701‐725.2057633210.1016/j.clnu.2010.05.004PMC71724122

Tomasik
PJ
, 
Tomasik
P
. Probiotics and prebiotics. Prof Nurs Today. 2012;15(3):12‐16,62.3

Kan
S
, 
Junko
K
, 
Rumi
K
, et al. Peptidoglycan from lactobacilli inhibits interleukin‐12 production by macrophages induced by *Lactobacillus casei* through Toll‐like receptor 2‐dependent and independent mechanisms. Immunology. 2009;128:e858‐e869.1974034710.1111/j.1365-2567.2009.03095.xPMC27538944

Livingston
M
, 
Loach
D
, 
Wilson
M
, et al. Gut commensal *Lactobacillus reuteri* 100–23 stimulates an immunoregulatory response. Immunol Cell Biol. 2010;88(1):99‐102.1978697910.1038/icb.2009.715

Lattemann
S
, 
Höpner
T
. Histamine derived from probiotic *Lactobacillus reuteri* suppresses TNF via modulation of PKA and ERK signaling. Desalination. 2012;7(2):e31951.10.1371/journal.pone.0031951PMC3285189223841116

Van Baalen
BP
, 
Troost
F
, 
Van der Meer
MC
, et al. Human mucosal in vivo transcriptome responses to three lactobacilli indicate how probiotics may modulate human cellular pathways. Proc Natl Acad Sci USA. 2011;108(Supplement_1):4562‐4569.2082323910.1073/pnas.1000079107PMC30635947

Lebeer
S
, 
Vanderleyden
J
, 
De Keersmaecker
SC
. Host interactions of probiotic bacterial surface molecules: comparison with commensals and pathogens. Nat Rev Microbiol. 2010;8(3):171‐184.2015733810.1038/nrmicro22978

Yasuda
M
, 
Jenne
CN
, 
Kennedy
LJ
, 
Reynolds
JD
. The sheep and cattle Peyer's patch as a site of B‐cell development. Vet Res. 2006;37:401‐415.1661155510.1051/vetres:20060089

Wesemann
DR
, 
Portuguese
AJ
, 
Meyers
RM
, et al. Microbial colonization influences early B‐lineage development in the gut lamina propria. Nature. 2013;501:112‐115.2396561910.1038/nature12496PMC380786810

Lanning
D
, 
Zhu
X
, 
Zhai
SK
, 
Knight
KL
. Development of the antibody repertoire in rabbit: gut‐associated lymphoid tissue, microbes, and selection. Immunol Rev. 2000;175:214‐228.10933605

## Supporting information

Figure S1

Figure S2

Figure S3

Figure S4

Figure Legends
