# Supplementary figures and images for: Effect of Lactobacillus rhamnosus on the development of B cells in gut‐associated lymphoid tissue of BALB/c mice
Source: J Cell Mol Med. 2020 Jul 8;24(15):8883–6. doi: 10.1111/jcmm.15574 (PMC7412698; doi:10.1111/jcmm.15574)

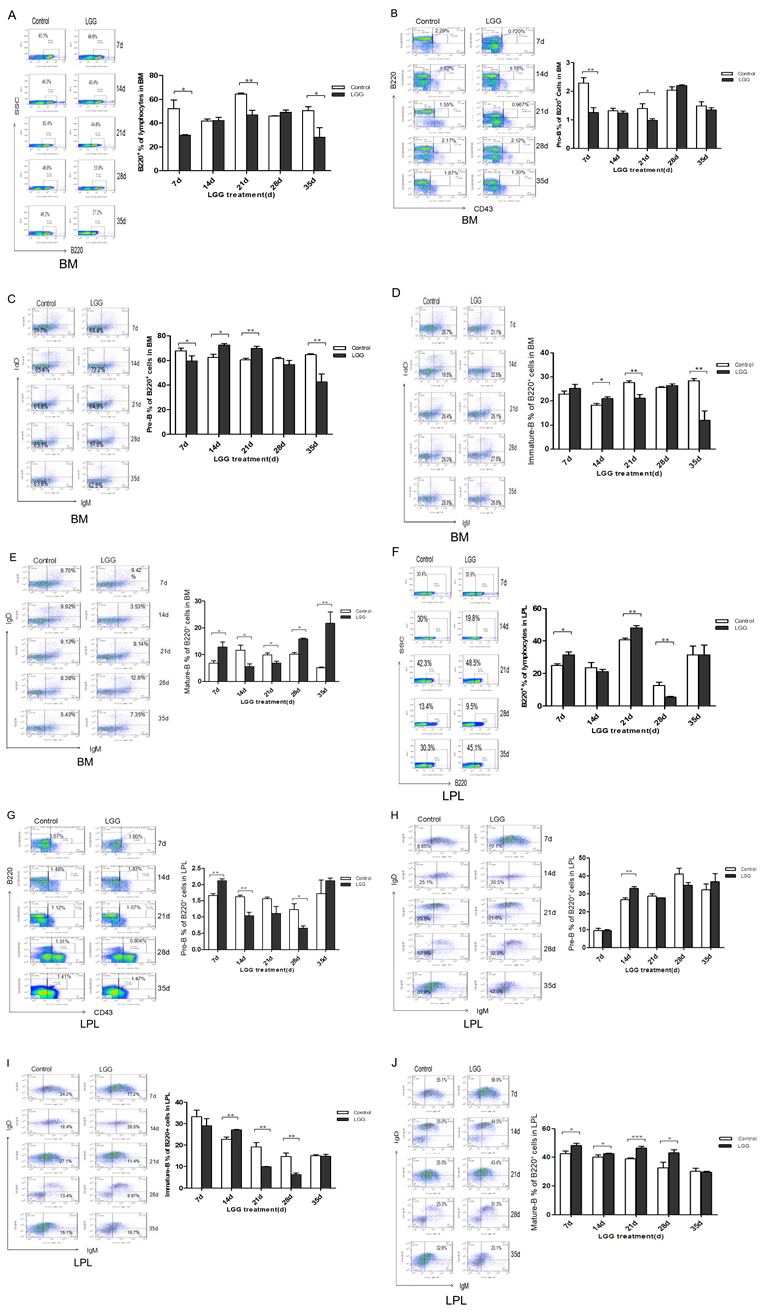

Supplement: Supplementary file 1 — Figure S1 [file JCMM-24-8883-s001.tif]

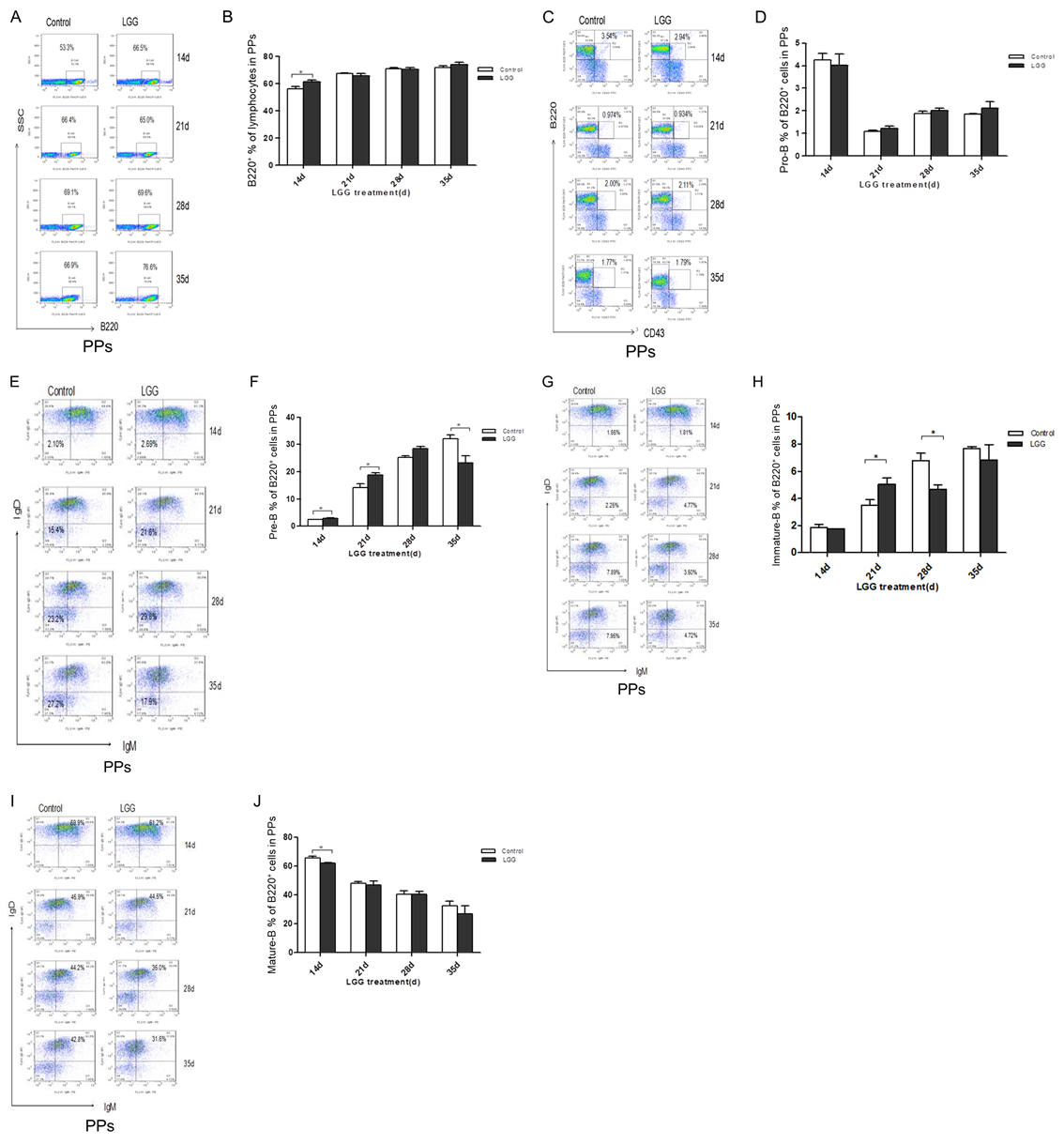

Supplement: Supplementary file 2 — Figure S2 [file JCMM-24-8883-s002.tif]

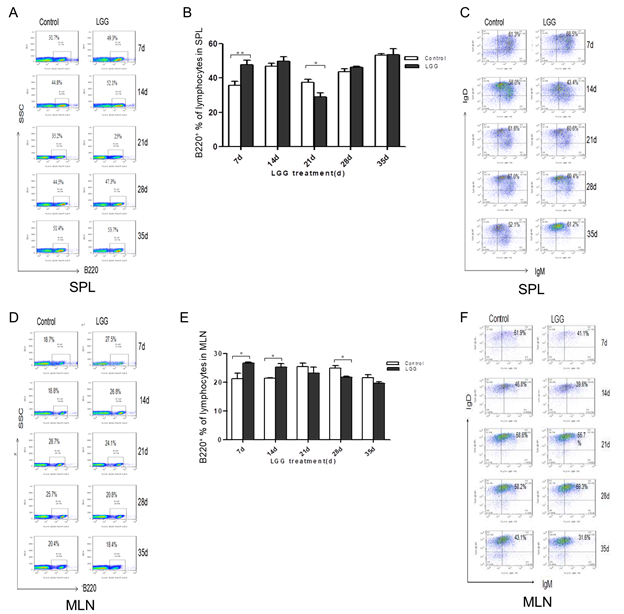

Supplement: Supplementary file 3 — Figure S3 [file JCMM-24-8883-s003.tif]

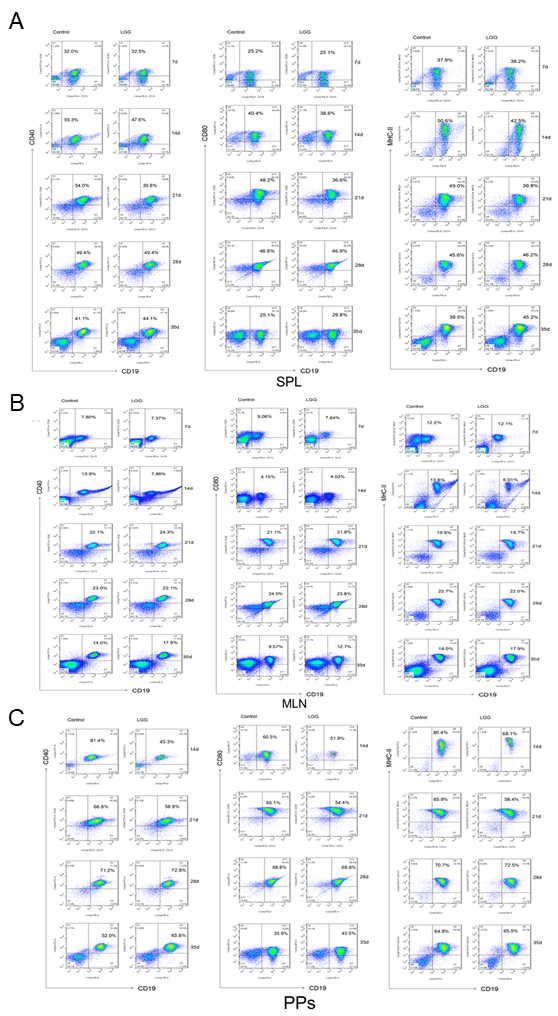

Supplement: Supplementary file 4 — Figure S4 [file JCMM-24-8883-s004.tif]
