# Supplementary material for: Effect of Lactobacillus rhamnosus on the development of B cells in gut‐associated lymphoid tissue of BALB/c mice
Source: J Cell Mol Med. 2020 Jul 8;24(15):8883–6. doi: 10.1111/jcmm.15574 (PMC7412698; doi:10.1111/jcmm.15574)
Supplement: Supplementary file 5 — Figure Legends [file JCMM-24-8883-s005.docx]

**[Figure S1.](sps:xml:id::jcmm15574-sup-0001||locator::supinfo\\jcmm15574-sup-0001-FigS1.tif||mimetype::image/tiff) The effect of LGG intervention on the development of B cells in BM and LPL.** (A) The proportion changes of total B cells (B220^+^) in the lymphocytes of BM with the LGG treatment. (B) The proportion changes of pro-B cells in the B220^+^ cells of BM with the LGG treatment. (C) The proportion changes of pre-B in the B220^+^ cells of BM with the LGG treatment. (D) The proportion changes of immature-B cells in the B220^+^ cells of BM with the LGG treatment. (E) The proportion changes of mature-B cells in the B220^+^ cells of BM with the LGG treatment. (F) The proportion changes of total B cells (B220^+^) in the lymphocytes of LPL with the LGG treatment. (G) The proportion changes of pro-B cells in the B220^+^ cells of LPL with the LGG treatment. (H) The proportion changes of pre-B in the B220^+^ cells of LPL with the LGG treatment. (I) The proportion changes of immature-B cells in the B220^+^ cells of LPL with the LGG treatment. (J) The proportion changes of mature-B cells in the B220^+^ cells of LPL with the LGG treatment. Student's t test was used to compare the data of the two groups, and multiple comparison method of one-way ANOVA was used to analyze the data of more than two groups. The symbol * indicated p<0.05, ** indicated p<0.01 and *** indicated p<0.001.

[**Figure S2.**](sps:xml:id::jcmm15574-sup-0002||locator::supinfo\jcmm15574-sup-0002-FigS2.tif||mimetype::image/tiff) **The effect of LGG intervention on the development of B cells in PPs.** (A-B) The proportion changes of total B cells (B220^+^) in the lymphocytes of PPs with the LGG treatment. (C-D) The proportion changes of pro-B cells in the B220^+^ cells of PPs with the LGG treatment. (E-F) The proportion changes of pre-B in the B220^+^ cells of PPs with the LGG treatment. (G-H) The proportion changes of immature-B cells in the B220^+^ cells of PPs with the LGG treatment. (I-J) The proportion changes of mature-B cells in the B220^+^ cells of PPs with the LGG treatment. Student's t test was used to compare the data of the two groups, and multiple comparison method of one-way ANOVA was used to analyze the data of more than two groups. The symbol * indicated p<0.05, ** indicated p<0.01 and *** indicated p<0.001.

[**Figure S3.**](sps:xml:id::jcmm15574-sup-0003||locator::supinfo\jcmm15574-sup-0003-FigS3.tif||mimetype::image/tiff) **The effect of LGG intervention on the proportion of total B cells and mature-B cells in secondary lymphoid organs.** (A-B) The proportion changes of total B cells (B220^+^) in the lymphocytes of SPL with the LGG treatment. (C) The representative data of mature-B cells in the B220^+^ cells of SPL with the LGG treatment. (D-E) The proportion changes of total B cells (B220^+^) in the lymphocytes of MLN with the LGG treatment. (F) The representative data of mature-B cells in the B220^+^ cells of MLN with the LGG treatment. Student's t test was used to compare the data of the two groups, and multiple comparison method of one-way ANOVA was used to analyze the data of more than two groups. The symbol * indicated p<0.05, ** indicated p<0.01 and *** indicated p<0.001.

[**Figure S4.**](sps:xml:id::jcmm15574-sup-0004||locator::supinfo\jcmm15574-sup-0004-FigS4.tif||mimetype::image/tiff) **The effect of LGG intervention on the activation and function of B cells.** (A) The representative data of CD19^+^ B cell expressing CD40/CD80/MHC-II among lymphocytes in SPL with the LGG treatment. (B) The representative data of CD19^+^ B cell expressing CD40/CD80/MHC-II among lymphocytes in MLN with the LGG treatment. (C) The representative data of CD19^+^ B cell expressing CD40/CD80/MHC-II among lymphocytes in PPs with the LGG treatment.
